# A Novel Method for Inducing Amastigote-To-Trypomastigote Transformation *In Vitro* in *Trypanosoma cruzi* Reveals the Importance of Inositol 1,4,5-Trisphosphate Receptor

**DOI:** 10.1371/journal.pone.0135726

**Published:** 2015-08-12

**Authors:** Muneaki Hashimoto, Jorge Morales, Haruki Uemura, Katsuhiko Mikoshiba, Takeshi Nara

**Affiliations:** 1 Department of Molecular and Cellular Parasitology, Juntendo University School of Medicine, 2-1-1 Hongo, Bunkyo-ku, Tokyo 113–8421, Japan; 2 Department of Protozoology, Institute of Tropical Medicine, Nagasaki University, 1-12-4 Sakamoto, Nagasaki 852–8523, Japan; 3 Laboratory for Developmental Neurobiology, RIKEN Brain Science Institute, Saitama 351–0198, Japan; 4 Calcium Oscillation Project, International Cooperative Research Project and Solution-Oriented Research for Science and Technology, Japan Science and Technology Agency, Kawaguchi, Saitama 332–0012, Japan; Tulane University, UNITED STATES

## Abstract

**Background:**

*Trypanosoma cruzi* is a parasitic protist that causes Chagas disease, which is prevalent in Latin America. Because of the unavailability of an effective drug or vaccine, and because about 8 million people are infected with the parasite worldwide, the development of novel drugs demands urgent attention. *T*. *cruzi* infects a wide variety of mammalian nucleated cells, with a preference for myocardial cells. Non-dividing trypomastigotes in the bloodstream infect host cells where they are transformed into replication-capable amastigotes. The amastigotes revert to trypomastigotes (trypomastigogenesis) before being shed out of the host cells. Although trypomastigote transformation is an essential process for the parasite, the molecular mechanisms underlying this process have not yet been clarified, mainly because of the lack of an assay system to induce trypomastigogenesis *in vitro*.

**Methodology/Principal Findings:**

Cultivation of amastigotes in a transformation medium composed of 80% RPMI-1640 and 20% Grace’s Insect Medium mediated their transformation into trypomastigotes. Grace’s Insect Medium alone also induced trypomastigogenesis. Furthermore, trypomastigogenesis was induced more efficiently in the presence of fetal bovine serum. Trypomastigotes derived from *in vitro* trypomastigogenesis were able to infect mammalian host cells as efficiently as tissue-culture-derived trypomastigotes (TCT) and expressed a marker protein for TCT. Using this assay system, we demonstrated that *T*. *cruzi* inositol 1,4,5-trisphosphate receptor (TcIP_3_R)—an intracellular Ca^2+^ channel and a key molecule involved in Ca^2+^ signaling in the parasite—is important for the transformation process.

**Conclusion/Significance:**

Our findings provide a new tool to identify the molecular mechanisms of the amastigote-to-trypomastigote transformation, leading to a new strategy for drug development against Chagas disease.

## Introduction

*Trypanosoma cruzi* is a parasitic protist that causes Chagas disease, which is prevalent in Latin America [1]. The life cycle of *T*. *cruzi* is complex and consists of two phases: insect and mammalian stages. Either stage includes replicative and infective (non-replicative) forms. In the insect stage, a replicative form called epimastigote dwells in the mid-gut of blood-sucking reduviid insects and transforms into the metacyclic trypomastigote (metacyclogenesis), which can infect mammalian hosts. In the mammalian stage, the metacyclic trypomastigote invades a wide variety of nucleated cells and transforms into an amastigote, which replicates in the host cell cytoplasm by binary fission. After successive rounds of replication, the amastigote transforms back into a trypomastigote, which disrupts the host cell and is released into the circulation, from where it can propagate the infection.

Transformation between the developmental stages is essential for parasite survival. Identification of the key factors that trigger transformation is important not only for understanding the nature of the parasite but also for developing anti-parasitic drugs. Among the transformation steps, the mechanisms underlying metacyclogenesis have been extensively studied. Metacyclogenesis is induced by free fatty acids [2], cAMP [3–5], and depletion of glucose [6] and is regulated via Ca^2+^ signaling [7,8]. Metacyclogenesis from cultured epimastigotes [9] can be reproduced *in vitro* by using Triatomine Artificial Urine (TAU) medium [10] and Modified Insect Medium [11].

Another transformation step reproducible *in vitro* is amastigote formation (amastigogenesis) from tissue-culture trypomastigotes by treatment with the acidic medium [12]. This treatment mimics the biological conditions where intracellular trypomastigotes transform into amastigotes in the acidic phagolysosome [13]. Amastigogenesis is regulated by Ca^2+^ signaling [7] and is associated with the ubiquitin-proteasome pathway [14–16]. More recently, a method for amastigogenesis from metacyclic trypomastigotes has been reported [17].

Trypomastigote formation from amastigotes (trypomastigogenesis) is crucial for generating an infective form in the mammalian stage. Several lines of evidence suggest that trypomastigogenesis involves protein-specific degradation via the proteasome [14] and requires proline in the proline auxotrophic CHO-K1 cell line [18]. However, despite the pathogenic importance of this step, its reproduction *in vitro* has not yet been successful. *In vitro* induction of trypomastigogenesis would shed light on the molecular mechanisms of this process.

In the present study, we report a novel method for inducing *in vitro* trypomastigogenesis from the *T*. *cruzi* extracellular amastigote. Trypomastigogenesis can be induced using the metacyclogenesis medium—a simple combination of commonly used culture media: 80% RPMI-1640 and 20% Grace’s Insect Medium. Using this system, we show that trypomastigogenesis is regulated via inositol 1,4,5-trisphosphate receptor (IP_3_R)-mediated Ca^2+^ signaling. Our findings provide a new experimental tool for the physiological investigation of this parasite and throw light on the pathogenic importance of Ca^2+^ signaling in *T*. *cruzi*.

## Methods

### *Trypanosoma cruzi* culture

Epimastigotes of *T*. *cruzi* Tulahuen strain were cultured as previously described [19]. *TcIP*_*3*_ *R*-SKO and EGFP-TcIP_3_R-overexpressing epimastigotes were cultured in liver infusion tryptose (LIT) medium (ATCC 1029) containing 0.25 mg/mL and 0.5 mg/mL G418, respectively [7]. Metacyclogenesis was performed as previously described [11]. Metacyclic trypomastigotes were added to the 3T3-Swiss Albino fibroblast cells (Health Science Research Bank, Tokyo, Japan), and the infected cells were passaged as described [20] until tissue culture trypomastigotes emerged in the culture supernatant. The tissue culture trypomastigotes were collected by centrifugation as described previously [20], and were thoroughly transformed into amastigotes by incubation with DMEM containing 0.4% BSA at pH 5.0 for 24 h, as described [12].

### Induction of amastigote-to-trypomastigote transformation

The parasites in the culture supernatant were collected by centrifugation at 1,600 × *g* for 10 min. The resulting cell pellet was re-suspended in 80% (v/v) RPMI-1640, HEPES medium (Life Technologies, Grand Island, NY, No. 22400–089) and 20% (v/v) Grace’s Insect Medium (Life Technologies, No. 11605–094). For some experiments, the previous formulation was supplemented with fetal bovine serum (FBS). All medium formulations were antibiotic-free. Amastigotes (1 × 10^7^) were incubated with 10 mL of the transformation medium in tightly capped 25-cm^2^ culture flasks at 26°C for predetermined periods. The number of amastigotes, intermediate forms, and trypomastigotes was determined by direct microscopic observation.

### Indirect fluorescent antibody assay (IFA)

The anti-*trans*-sialidase monoclonal antibody (MAb 39) was provided by Dr. Sergio Schenkman (Universidade Federal de São Paulo, Brazil). IFA was performed as previously described [7].

### Statistical analysis

Statistical analysis was performed with Sigma Plot ver. 1.2 software (Systat Software, Inc., Sun Jose, CA) using one-way ANOVA and Fisher’s PLSD post-hoc test.

## Results and Discussion

### Incubation of amastigotes in the metacyclogenesis medium results in the induction of trypomastigote transformation

**Fig 1:**
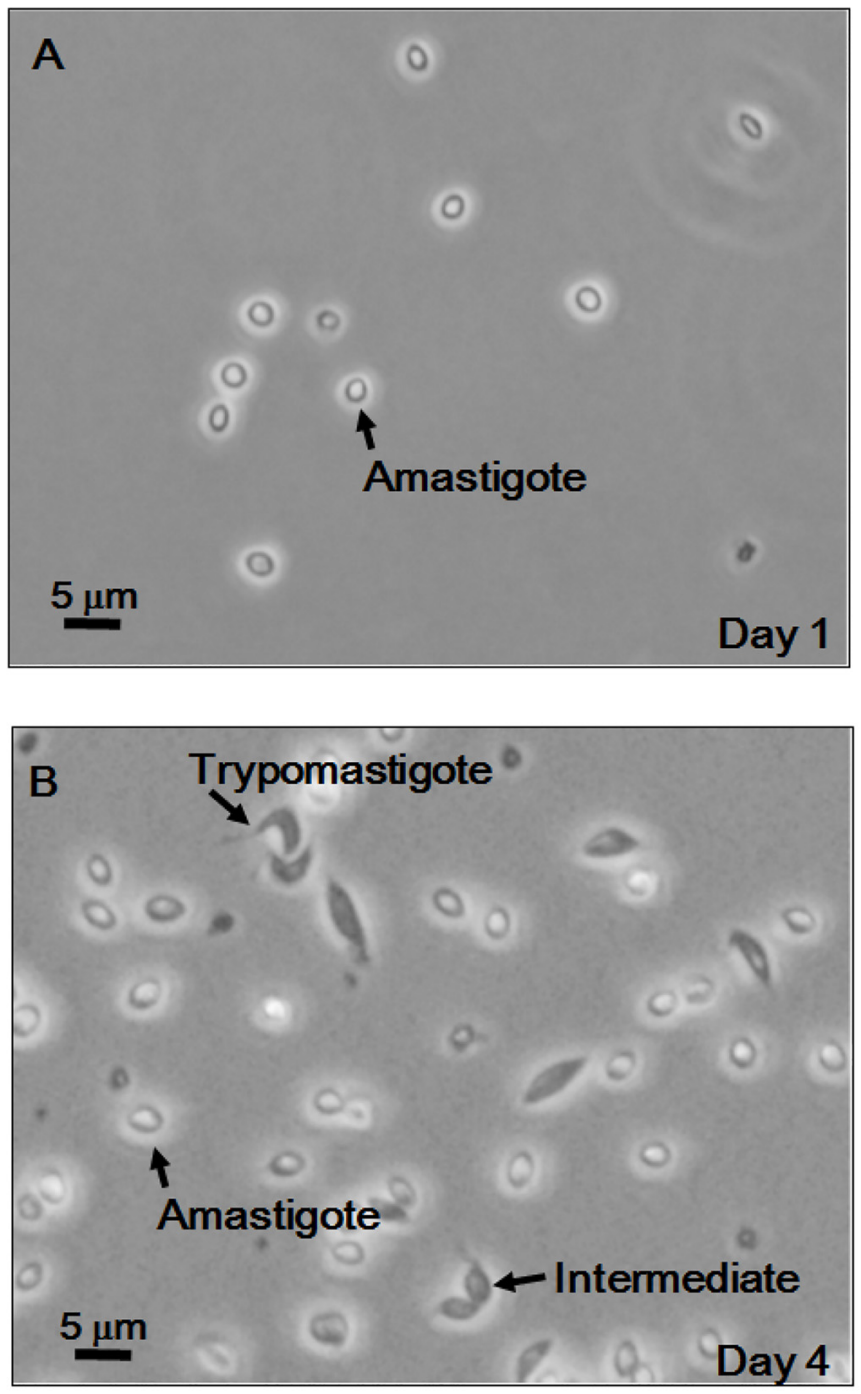
Microscopic images of amastigote-to-trypomastigote transformation. Amastigotes (1 × 10^6^ cells/mL) were cultured in 80% RPMI-1640 plus 20% Grace’s Insect Medium for 1 day (A) or 4 days (B). Representative microscopic images are shown by using an inverted light compound microscope (Olympus, IX71, Tokyo, Japan) with 60x objective. A video of the same field is available in S1 Movie.

Biologically, epimastigotes resemble amastigotes; both forms can replicate by binary fission and are able to transform into their respective infective forms: metacyclic trypomastigote and bloodstream trypomastigote. Therefore, we hypothesized that trypomastigogenesis may be triggered *in vitro* in a manner similar to metacyclogenesis [11,21]. Amastigote preparations used for the experiments were easily obtained by treating the tissue-culture trypomastigotes with acidic buffer at 37°C for 24 h (Fig 1A) [12].

We first tested whether the metacyclogenesis medium (80% RPMI-1640 plus 20% Grace’s Insect Medium) could induce trypomastigogenesis. As expected, incubation of extracellular amastigotes in the metacyclogenesis medium at a cell density of 1 × 10^6^ cells/mL at 26°C for 4 days led to the generation of the flagellated form of the parasite (Fig 1B). The culture contained fully developed trypomastigotes and an intermediate form, the latter of which looked amastigote but had an obvious flagellum (shown as “Intermediate” in Fig 1B). In contrast, the resulting trypomastigotes were very motile and looked rather short and stumpy (shown as “Trypomastigote” in Fig 1B. See also S1 Movie). This morphology was frequently observed in tissue-culture trypomastigotes compared to metacyclic trypomastigotes. These results suggest that our method can successfully induce trypomastigogenesis *in vitro* for the first time.

Unexpectedly, although trypomastigogenesis occurrs in the host cells at 37°C, the incubation of amastigotes at 37°C did not effectively induce trypomastigogenesis. The reasons for this phenomenon are unknown. However, since trypomastigotes derived from *in vitro* trypomastigogenesis are biologically tissue-cultured trypomastigotes as described below, our findings may contribute to the understanding of the molecular mechanisms involved in trypomastigogenesis as well as the extension of a flagellum from an amastigote.

### Grace’s Insect Medium is essential for trypomastigogenesis

**Fig 2:**
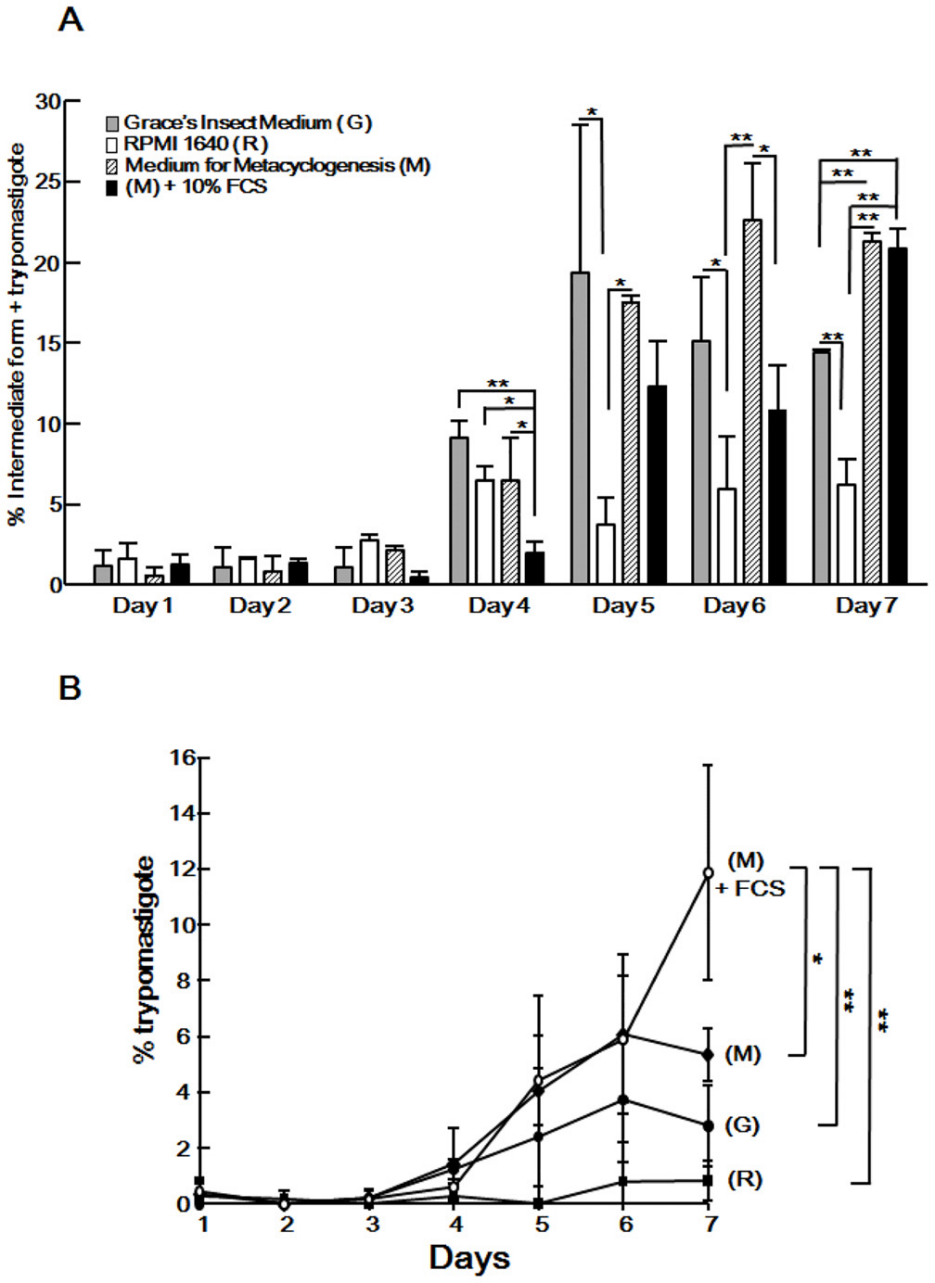
Optimization of the medium for amastigote-to-trypomastigote transformation. (A) Amastigotes (1 × 10^6^ cells/mL) were cultured in Grace’s Insect Medium, RPMI-1640 medium, 80% RPMI-1640 plus 20% Grace’s insect medium, or 70% RPMI-1640 plus 20% Grace’s insect medium containing 10% FBS for 7 days. The histogram depicts the percentage of intermediate forms and trypomastigotes, which was calculated by daily counting of the different forms by using IX71 with 60 x objective. More than 100 parasites were randomly counted. (B) The percentage of trypomastigotes is shown in Fig 2A. Data shown are the mean ± S.D. of 3 independent experiments. *P < 0.05, **P < 0.01.

We next investigated the optimal conditions for *in vitro* trypomastigogenesis (Fig 2). In the metacyclogenesis medium, amastigotes began to sprout flagella at day 4 post-incubation. The maximum efficiency of flagellation was about 20% (including an intermediate form, see also Fig 1) and obtained at day 6 (Fig 2A). Longer incubation periods (>7 days) led to the death of the parasites.

We further examined the effect of the composition of the incubation medium on trypomastigogenesis. To investigate whether the individual components of the metacyclogenesis medium could induce trypomastigogenesis, amastigotes were incubated in either Grace’s medium or RPMI-1640. Notably, Grace’s medium alone could induce a comparable level of trypomastigogenesis to that induced by the metacyclogenesis medium by day 6. At day 7, we observed more dead parasites in Grace’s medium and the transformation efficiency was significantly lower than in the metacyclogenesis medium. These data suggest that Grace’s medium contains the essential factor(s) for trypomastigogenesis but it does not support the longer period required for parasite survival. In contrast, RPMI-1640 did not support efficient transformation of amastigotes. Furthermore, we found that the addition of fetal calf serum (FCS) to the metacyclogenesis medium (70% RPMI-1640; 20% Grace’s medium; 10% FBS) significantly increased the level of trypomastigote formation at day 7 (*P*<0.05, Fig 2B). We designated this medium as “trypomastigogenesis medium” and used it for further experiments. On the other hand, it is striking at day 4 and day 6 that the trypomastigogenesis medium significantly inhibited the transformation of amastigotes compared to the metacyclogenesis medium (Fig 2A). Since the metacyclogenesis medium induced a comparable level of trypomastigogenesis to that induced by the trypomastigogenesis medium by day 6 (Fig 2B), FCS may inhibit flagella extension of amastigotes. However, for longer periods of cultivation, FCS may supply nutrients for the parasites in order to produce trypomastigotes effectively.

### Trypomastigotes derived from in vitro trypomastigogenesis are biologically tissue-culture-derived trypomastigotes

**Fig 3:**
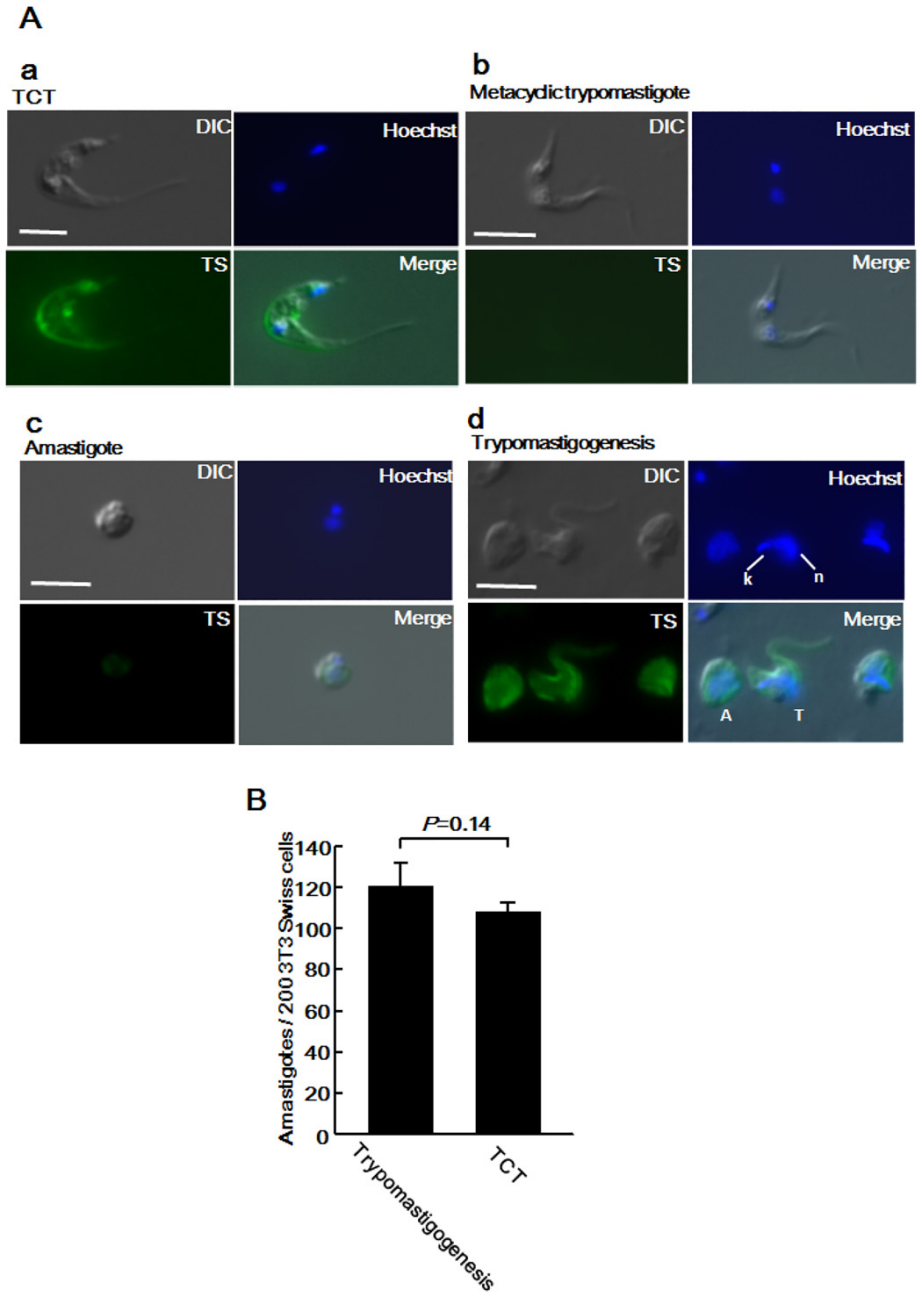
Biological properties of trypomastigotes derived from *in vitro* trypomastigogenesis. (A) Tissue-culture trypomastigotes (a), metacyclic trypomastigotes (b), amastigotes (c), or the parasites cultured for 6 d in trypomastigogenesis medium (d) were fixed, incubated with anti-*trans*-sialidase antibody, and stained with Alexa Fluor 488-labelled (green) secondary antibody. The nuclei (n) and kinetoplast (k) (blue) were counter-stained using Hoechst 33342. (B) Trypomastigotes derived from trypomastigogenesis or tissue-culture trypomastigotes (2 × 10^5^) were incubated with 2 × 10^4^ 3T3-Swiss albino cells for 12 h. For calculation of the infectivity, the number of intracellular parasites in a total of 200 cells was counted after Giemsa staining. Data shown are the mean ± S.D. of 3 independent experiments. Statistical analysis between the groups was performed using Student’s *t*-test.

We compared the properties of trypomastigotes derived from trypomastigogenesis to those of tissue-culture-derived trypomastigotes (Fig 3). Tissue-culture-derived trypomastigotes express strong *trans*-sialidase activity, but metacyclic trypomastigotes express very little *trans*-sialidase activity [22]. The protein structures of these trans-sialidases have similar N-terminal catalytic domains but their C-terminal regions are different; the former containing 12 amino acid repeats and the latter lacking this repeat sequence. These can be distinguished by monoclonal antibody 39 (Mab 39), which recognizes the C-terminal repeat region of trypomastigote-specific trans-sialidase [23]. An indirect fluorescent antibody assay (IFA) with the anti-*trans*-sialidase antibody (MAb 39) was performed against tissue-culture trypomastigotes (Fig 3Aa) and metacyclic trypomastigotes (Fig 3Ab). Expression of *trans*-sialidase containing C-terminal repeats was detected only in tissue-culture trypomastigotes. IFA with MAb 39 was then performed against the parasites derived from trypomastigogenesis. The trypomastigotes expressed trypomastigote-specific *trans*-sialidase (Fig 3Ad, T). The amastigotes in trypomastigogenesis medium also expressed *trans*-sialidase after 6 d of incubation (Fig 3Ad and A) but not before the induction of trypomastigogenesis (Fig 3Ac). The intracellularly dividing amastigotes were not found to react with Mab 39 according to previous reports [23], but trans-sialidase enzyme activity was detected in fully matured amastigotes just before trypomastigotes were observed in the host cell. The Mab 39-positive amastigotes observed in the trypomastigogenesis medium might be similar to these parasites.

Next, we investigated whether trypomastigotes derived from *in vitro* trypomastigogenesis actually infect mammalian host cells (Fig 3B). Trypomastigotes derived from trypomastigogenesis and tissue-culture trypomastigotes were incubated with 3T3-Swiss albino cells for 12 h, and the number of infected amastigotes was counted. The number of infected amastigotes was not significantly different between the two groups (*P* = 0.14). In these experiments, the culture medium was incubated for 30 min in tubes after trypomastigogenesis before being used to infect the host cells. Since amastigotes do not swim due to the lack of flagella, most amastigotes remained at the bottom of the tubes. We recovered the trypomastigotes from the supernatant and found that the number of contaminating amastigotes was very few (data not shown). Furthermore, extracellular amastigotes of type I *T*. *cruzi* (*e*.*g*. G strain) efficiently invade into non-professional phagocytes, but type II *T*. *cruzi* have a limited ability to invade this cell type [24]. Since our studies utilized the Tulahuen type II *T*. *cruzi* strain [25], the possibility of infection of the extracellular amastigotes into the host cells was negligible. Taken together, these results indicate that trypomastigotes derived from *in vitro* trypomastigogenesis are biologically tissue-culture-derived trypomastigotes.

### TcIP_3_R regulates trypomastigogenesis

IP_3_R is an intracellular Ca^2+^ channel that releases Ca^2+^ upon various stimuli via activation by IP_3_. We previously reported that Ca^2+^ signaling via *T*. *cruzi* IP_3_R (TcIP_3_R) controls a variety of the parasitic biological processes, including metacyclogenesis and amastigogenesis [5]. That is, reduced and increased levels of TcIP_3_R expression promote metacyclogenesis and amastigogenesis, respectively. In the present study, we were interested to know whether TcIP_3_R-mediated Ca^2+^ signaling is associated with trypomastigogenesis in the present setup (Fig 3). For phenotypic analysis, we used TcIP_3_R-knockdown and TcIP_3_R-overexpressing *T*. *cruzi*. For the TcIP_3_R-knockdown construct, we had previously established TcIP_3_R-single knockout (SKO) parasites in which one of three *TcIP*_*3*_ *R* gene loci was disrupted and the level of *TcIP*_*3*_ *R* transcript was reduced to two-thirds of that in wild type (WT). For TcIP_3_R overexpression, we used *T*. *cruzi* expressing N-terminally EGFP-tagged-TcIP_3_R (EGFP-TcIP_3_R) [7].

**Fig 4:**
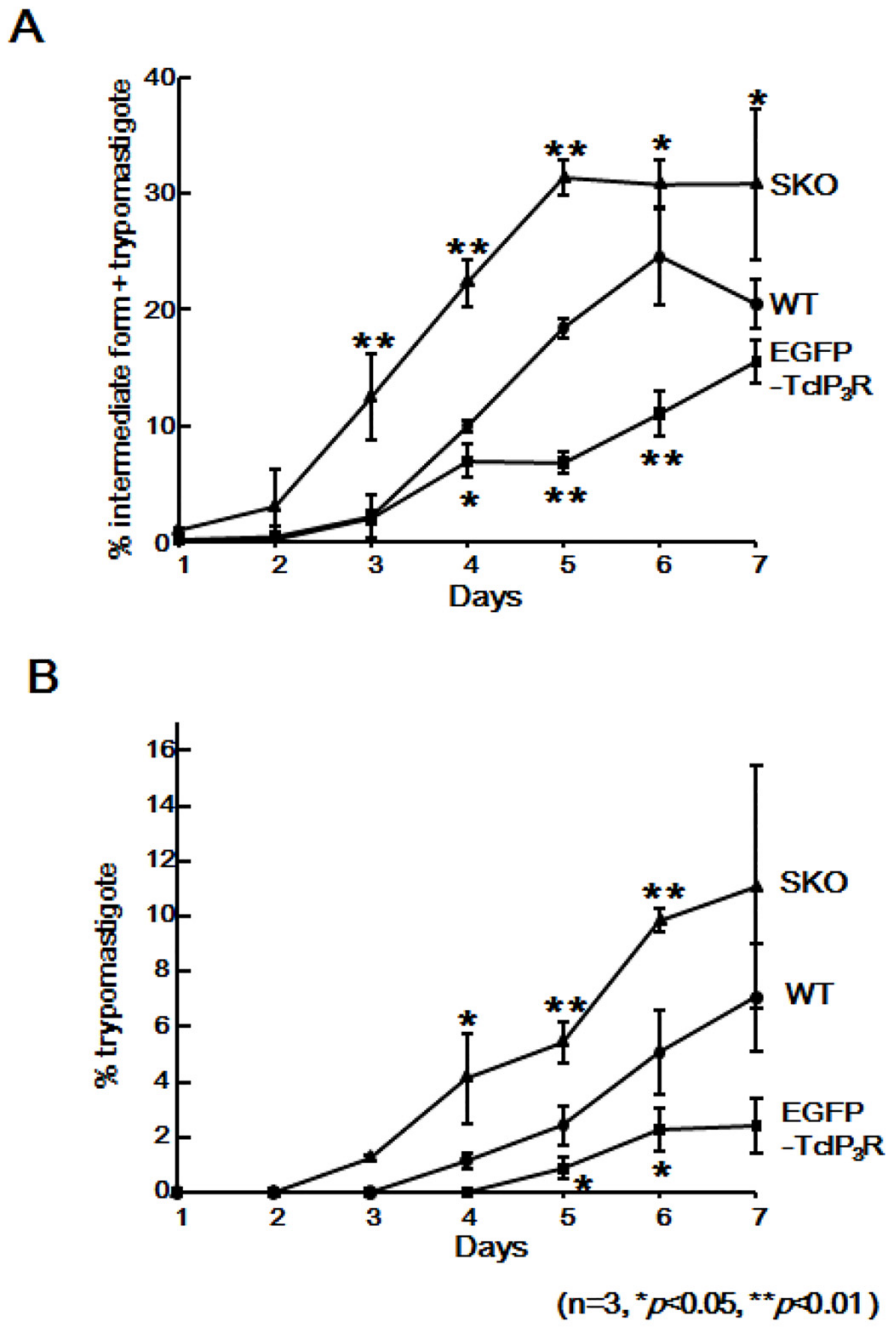
Involvement of TcIP_3_R in amastigote-to-trypomastigote transformation. (A) Amastigotes of WT or *TcIP*_*3*_ *R*-SKO- or EGFP-TcIP_3_R-overexpressing parasites were cultured in a medium composed of 70% RPMI-1640 and 20% Grace’s Insect Medium supplemented with 10% FCS for 7 days. The percentage of the intermediate forms plus trypomastigotes was calculated daily. (B) The percentage of trypomastigotes is shown in Fig 3A. Data shown are the mean ± S.D. of 3 independent experiments. *P < 0.05, **P < 0.01.

We compared the efficiency of trypomastigogenesis between WT, SKO, and EGFP-TcIP_3_R cells at all days. We found a significantly larger population of flagellated parasites (intermediate + trypomastigotes) in the SKO parasites, whereas EGFP-TcIP_3_R parasites showed the lowest transformation levels of all three (Fig 4A). Similarly, the population of trypomastigotes was significantly higher in the SKO parasites and lower in those of EGFP-TcIP_3_R than that of WT (Fig 4B). These results suggest that TcIP_3_R-mediated Ca^2+^ signaling plays a pivotal role in trypomastigogenesis, metacyclogenesis, and amastigogenesis, and that the reduced levels of Ca^2+^ release via TcIP_3_R is likely to drive the transformation into the infective state in *T*. *cruzi*.

Besides trypanosomatids, Ca^2+^ signaling is essential for the survival of other parasitic protists. For example, Ca^2+^ regulates a variety of vital functions such as motility, cell invasion, protein secretion, and differentiation in apicomplexan parasites including *Plasmodium*, *Toxoplasma* and *Cryptosporidium* [26–34]. Thus, Ca^2+^ appears to be critical for the survival of a range of parasitic protists, and our data indicate that Ca^2+^ signaling influences multiple developmental stages of *T*. *cruzi*

In the present study, we developed a novel and simple method to induce trypomastigogenesis *in vitro*. Although our research has provided the framework for generating trypomastigotes *in vitro*, we were unable to fully reproduce the robustness of trypomastigogenesis that occurs in mammalian host cells. First, only 10–15% of extracellular amastigotes incubated in trypomastigogenesis medium for 7 days transformed into tissue-culture trypomastigotes (Fig 2). Considering that almost all amastigotes in mammalian cells typically transform into trypomastigotes in 24 h, the rate of transformation *in vitro* is very low. One possible explanation for this discrepancy is that the parasites become weak due to the *in vitro* incubation with acidic medium for long durations. Additionally, trypomastigogenesis in mammalian cells occurs at 37°C, but the *in vitro* transformation is induced at 26°C more effectively. These results indicate that other factor(s) are required to reproduce trypomastigogenesis *in vitro* to the level of that which occurs in mammalian cells. However, our method provides important insights into the molecular cues underlying trypomastigogenesis, a process of great pathogenic importance in mammalian hosts. Furthermore, our method provides targets for inhibitory compounds of trypomastigogenesis, which is a critical step for parasite survival. We also show that TcIP_3_R-mediated Ca^2+^ signaling plays a fundamental role in the multiple differentiation steps of *T*. *cruzi*. Further analysis is necessary to identify the stimulating molecules that promote IP_3_ synthesis, leading to Ca^2+^ signaling in the different developmental stages of the parasite.

## Supporting Information

S1 Movie Microscopic movie of amastigote-to-trypomastigote transformation. Amastigotes (1 x 10^6^ cells/mL) were cultured in 80% RPMI-1640 plus 20% Grace’s Insect Medium for 4 days. The field of the video is identical to that of Fig 1B. Note that the trypomastigote is very motile and have an obvious flagellum.
(M4V)
